# Variations of Alkaloid Accumulation and Gene Transcription in *Nicotiana tabacum*

**DOI:** 10.3390/biom8040114

**Published:** 2018-10-15

**Authors:** Bo Sun, Yu-Xiao Tian, Fen Zhang, Qing Chen, Yong Zhang, Ya Luo, Xiao-Rong Wang, Fu-Cheng Lin, Jun Yang, Hao-Ru Tang

**Affiliations:** 1 College of Horticulture, Sichuan Agricultural University, Chengdu 611130, China; 14099@sicau.edu.cn (B.S.); yuxiao_tian@stu.sicau.edu.cn (Y.-X.T.); zhangf_12@163.com (F.Z.); supnovel@gmail.com (Q.C.); zhyong@sicau.edu.cn (Y.Z.); luoya945@163.com (Y.L.); wangxr@sicau.edu.cn (X.-R.W.); 2 Zhengzhou Tobacco Research Institute, Zhengzhou 450001, China; fuchenglin@zju.edu.cn

**Keywords:** *Nicotiana tabacum*, alkaloids, gene transcription, developmental stages, varieties

## Abstract

To increase the understanding of alkaloid biosynthesis in *Nicotiana tabacum* during whole plant growth periods, variations of the contents of alkaloids and the transcription of key biosynthetic genes in fresh leaves were investigated in three varieties at five developmental stages. Six alkaloids were analyzed by gas chromatograph–mass spectrometry (GC–MS) and the most abundant alkaloid was observed during the upper leaves maturing stage in the varieties, among which the alkaloid content of K326 was the highest. Considering the genetic effect, variance analysis indicated that the developmental stage played a predominant role in alkaloid accumulation. Moreover, the levels of biosynthetic gene transcripts in the leaves at the vigorous growing stage might contribute to the contents of alkaloids in the leaves during the maturing stages. To further illuminate the metabolism of alkaloid biosynthesis, a correlation among alkaloids was also documented.

## 1. Introduction

Alkaloids are one of the most diverse groups of secondary compounds found in plants and consist of low molecular weight, nitrogenous substances [1,2]. Approximately 20% of plant species accumulate alkaloids, which are mostly derived from amino acids, including phenylalanine, tyrosine, tryptophan, lysine, and ornithine [1]. Since the identification of the first alkaloid, morphine, from *Papaver somniferum*, by Sertȕrner in 1806, over 12,000 different alkaloids have been described, indicating their structural and biosynthetic diversity compared to that of other secondary metabolites [1,3]. Alkaloids are known to function in the chemical defense of plants against herbivores and pathogens. They have been also exploited as pharmaceuticals, stimulants, and narcotics because of their pronounced and various physiological activities in animals and humans [3,4,5].

**Figure 1:**
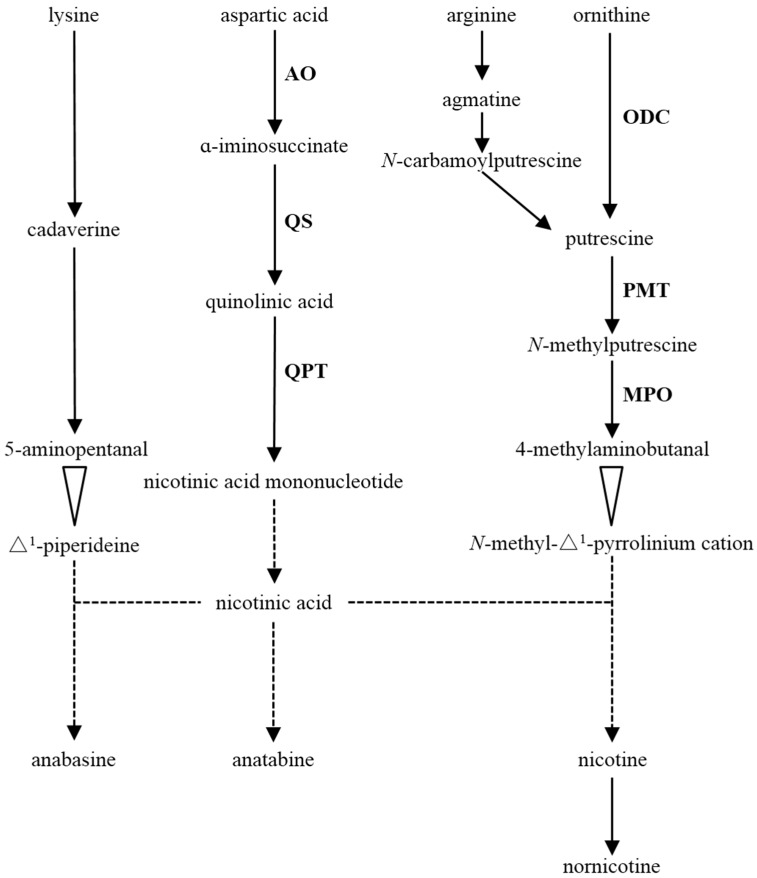
Alkaloid biosynthetic pathways in *Nicotiana tabacum* (modified from Häkkinen, 2007 [9] and Shoji 2010 [4]). Solid arrows, dashed arrows, and white arrowheads represent enzymatic reactions defined biochemically, undefined steps, and spontaneous, respectively. The enzymes examined in this study are abbreviated as follows: AO, aspartate oxidase; MPO, *N*-methylputrescine oxidase; ODC, ornithine decarboxylase; PMT, putrescine *N*-methyltransferase; QPT, quinolinic acid phosphoribosyl transferase; QS, quinolinic acid synthase.

*Nicotiana tabacum* contains a number of structurally related alkaloids [6] and is considered as the model plant for alkaloid research. Nicotine is the predominant alkaloid accumulating in the leaves of most *N. tabacum* varieties and represents 90–95% of the total alkaloid content [2,4,7,8]. Since the 1970s, plenty of enzymes in alkaloid biosynthesis pathways have been discovered and partially characterized (Figure 1) [3]. Nicotine biosynthesis begins with putrescine, which can be synthesized directly from ornithine by ornithine decarboxylase (ODC) and/or formed indirectly from arginine by arginine decarboxylase (ADC). Putrescine is converted to *N*-methylputrescine by the action of putrescine *N*-methyltransferase (PMT), which is the first committed step in nicotine biosynthesis [2,7]. *N*-methylputrescine is then oxidized by *N*-methylputrescine oxidase (MPO) and cyclized to form the pyrrolidine ring. In addition, aspartate oxidase (AO), quinolinic acid synthase (QS), and quinolinic acid phosphoribosyl transferase (QPT) serve in pyridine ring synthesis that supplies nicotinic acid [2,4,9].

As one kind of important secondary metabolites, alkaloids in *N. tabacum* have been widely investigated in previous studies [2,4,7,8,10]. However, the synthesis and accumulation of alkaloids are controlled by various genetic, developmental, environmental factors and other cues, instead of a random process [1,2]. Although the variation of alkaloids among different varieties has been surveyed using *N. tabacum* baked leaves [10,11,12], there is little information on alkaloid profiles in fresh leaves during different developmental stages so far. Previous studies have shown that nicotine was mainly synthesized in the roots and transported to the shoots through the xylem [7,13]. Nevertheless, only limited information on the connection between the content of alkaloids and their structural gene transcription patterns in leaves is available. Therefore, the objectives of this study were to evaluate the composition and content of alkaloids, as well as the transcription levels of key biosynthetic genes in fresh leaves during five developmental stages. In addition, genetic effects and correlation between different alkaloids were also documented. Our systematic study will contribute to the elucidation of the mechanisms of alkaloid metabolism and regulation.

## 2. Materials and Methods

### 2.1. Plant Materials

Three main varieties of *N. tabacum* in China, cv. Hongda, K326 and Zhongyan 100 were planted in the most suitable site for each in our experiments (Table 1). The altitude, annual sun exposure time, annual precipitation, and annual average temperature of the planting region in Xiangyun county were 1800 m above sea level, 2327 h, 810 mm, and 14.7 °C, those in Zunyi county were 1300 m above sea level, 1177 h, 1036 mm, and 14.9 °C, those in Xiangxian county were 90 m above sea level, 2280 h, 700 mm, and 14.7 °C, respectively. The data were obtained from the China Meteorological Administration. In February 2011, the sterilized seeds of each variety germinated and were grown in a floating system in a greenhouse with a 14 h light and 10 h dark photoperiod and an average temperature cycle of 25/20 °C. After 70 days, 200 seedlings of each variety with 7–9 true leaves were transplanted into an agricultural field with a row distance of 120 cm and an individual plant distance of 60 cm. The field design was completely random. Water, fertilizer, and pesticides were applied as necessary.

Plant materials free of any insects and mechanical damage were harvested at five different developmental stages, i.e., the rosette stage, the vigorous growing stage, the lower leaves maturing stage, the middle leaves maturing stage, and the upper leaves maturing stage. The leaves at the specific positions at different growth periods were the target sample materials. Three plants with a similar growing tendency were grouped as a replicate and there were three replicates for each sample. The plant sites, sampling time and leaf position carried out in this experiment are described in Table 1.

Samples were immediately placed into liquid nitrogen and transported instantly to the laboratory of Zhengzhou Tobacco Research Institute (ZTRI) in a box filled with dry ice. Parts of the samples were lyophilized with a freeze dryer (VirTis Inc., New York, NY, USA) and the lyophilized samples were ground into a fine powder using a coffee mill and stored at −20 °C for alkaloid analyses, while the remainder was stored at −80 °C and used for quantitative real-time polymerase chain reaction (qRT-PCR) analyses.

### 2.2. Alkaloid Analysis

Alkaloids were extracted from lyophilized samples and analyzed as previously described with a minor modification [14]. A 300 mg sample of leaf powder was added to 2.0 mL of 5% NaOH in a 50 mL conical flask and the mixture was incubated for 15 min at room temperature. Alkaloids were extracted by the addition of 20 mL of extraction solution [180 mg L^−1^ 2-methylquinoline (TCI, Tokyo, Japan) and 10 mg L^−1^ 2,4′-bipyridyl (TCI) dissolved in 0.01% triethylamine (Fluka, Newport News, VA, USA)]/chloroform (Merck, Darmstadt, Germany) and ultrasonically extracted for 15 min at 20 °C. Following the phase separation, an aliquot of the organic phase was filtered through a column filled with anhydrous sodium sulfate. Two milliliter of the filtrate was transferred to a sample vial and subjected to gas chromatograph–mass spectrometry (GC–MS) analysis.

Qualitative and quantitative analyses of alkaloids in *Nicotiana tabacum* leaves were performed using an Agilent 7890A gas chromatograph (GC) interfaced with an Agilent 5975C mass-selective detector (Agilent Technologies Inc., Santa Clara, CA, USA), which was controlled by an Agilent G1701EA GC–MSD ChemStation. The GC was equipped with an HP-35 capillary column (Agilent Technologies Inc., Santa Clara, CA, USA), 30 m × 0.250 mm I.D. and a 0.25 μm film thickness. Helium was used as a carrier gas with a flow of 1.0 mL min^−1^. The temperature of the oven was set initially at 100 °C for 3 min and it was programmed to increase at 8 °C min^−1^ to 260 °C and finally held for 10 min. The injector temperature was 250 °C set at split mode (10:1) with an injection volume of 2 μL.

The mass detector was operated with electron impact ionization (EI, 70 eV) with an ion source temperature of 230 °C and an interface temperature of 280 °C. The mass spectrometer was set to full-scan mode (mass range *m*/*z* 30–500 for qualitative analysis of alkaloids) and selected ion monitoring (SIM) mode was used for quantitative analysis. Ions were acquired in SIM mode with a solvent cut time of 8.0 min.

### 2.3. Quantitative Real Time PCR Analysis

For isolation of total RNA, leaf samples were ground and homogenized in liquid nitrogen. Total RNA was isolated using the RNeasy Plant Mini kit (Qiagen, Hilden, Germany) according to the manufacturer’s instructions. The RNA concentration and quality were assessed by photometric measurement (GE, Livingston, NJ, USA) and gel electrophoresis. Approximately 2 μg of total RNA was utilized to synthesize complementary DNA (cDNA) with avian myeloblastosis virus (AMV) reverse transcriptase (Takara, Kusatsu, Japan) and Oligo (dT)_18_ primers, as described in the manufacturer’s instructions (Takara, Kusatsu, Japan).

cDNA templates were amplified using a Bio-Rad iCycler thermocycler (Bio-Rad, Hercules, CA, USA) with SYBR Premix EX Taq (Takara). A total reaction volume of 20 μL contained 10 μL 2 × SYBR Premix EX Taq, 2 μL of diluted cDNA and 1 μL of each gene-specific primer (10 μM). The primer sequences are listed in Table 2. The protocol of the qRT-PCR was performed as follows: 95 °C for 5 min; 60 cycles of 95 °C for 10 sec, 55 °C for 10 sec, 72 °C for 10 sec followed by final extension at 72 °C for 5 min [4]. The specificity of the reactions was confirmed by the machine’s standard melt curve method. The data were expressed as the final cycle number necessary to reach a threshold fluorescence value (C_t_), and normalized by the 2^−ΔΔCt^ method [15]. 26S-RNA was used as a reference gene [16]. Each assay was repeated at least three times.

### 2.4. Statistical Analysis

The genetic model including genotype × environment interactions developed by Zhu was used for the analysis of inheritance [17]. The model used for the analysis was:

*Y_ijk_* = *μ* + *G_i_* + *E_j_* + *GE_ij_* + *e_ijk_*

where *Y_ijk_* = the phenotypic mean of the cross of variety *i* and developmental stage *j* in the *k*th block; *μ* = population mean; *G_i_* = the variety effect; *E_j_* = the developmental stage effect; *GE_ij_* = the variety × developmental stage interaction effect; *e_ijk_* = the residual error.

The resultant data were analyzed with the TestR Model by the minimum norm quadratic unbiased estimation (MINQUE) method in QGAStation Version 2.0 software (Institute of Bioinformatics, Zhejiang University, Hangzhou, China) for estimating variances and covariances and further calculating the ratios of genetic variance over phenotypic variance [18]. The Jackknife resampling method was used to calculate the standard errors of the estimated values for the *t*-test and significant differences were tested by the *t*-test [19].

Statistical analyses were performed via the SPSS package program version 11.5 (SPSS Inc., Chicago, IL, USA). Data were analyzed with the one-way ANOVA model. The variants were variety and developmental stage. The means were compared through the least significant differences (LSD) test at a significance level of 0.05. The values were reported as means with standard deviations.

## 3. Results and Discussion

### 3.1. The Composition and Content of Alkaloids

**Figure 2:**
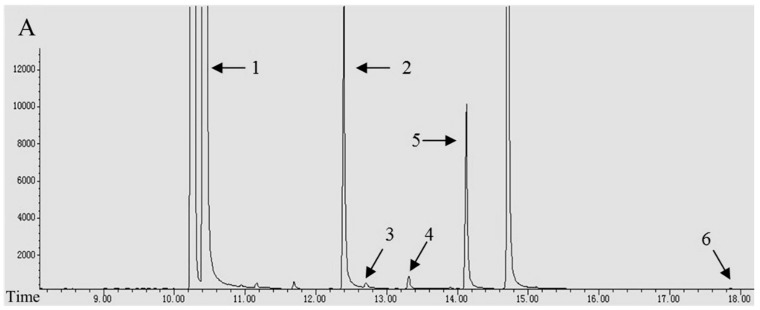

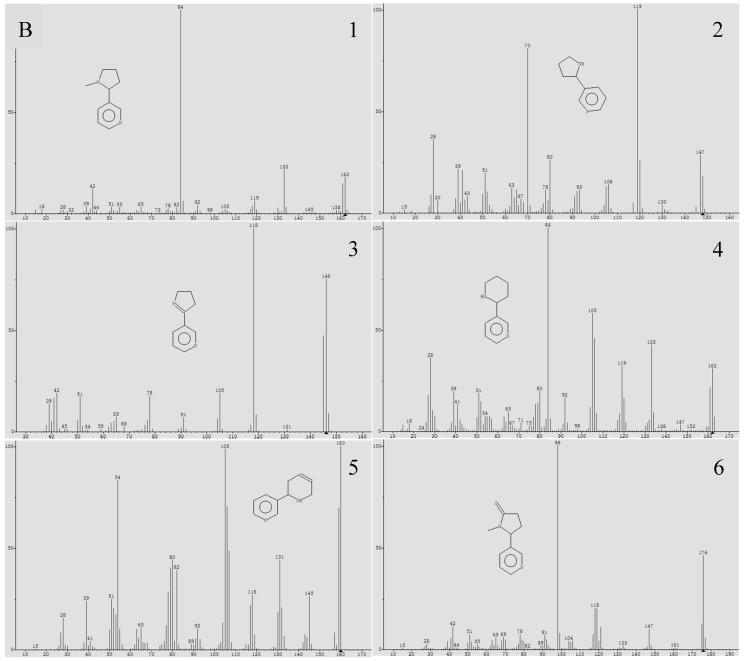
Gas chromatography-selected ion monitoring mode-mass spectrometry (GC–SIM-MS) chromatogram of alkaloids in *Nicotiana tabacum*. (**A**) Total ion current (TIC) chromatogram of alkaloids in *Nicotiana tabacum*. Peaks: (1) nicotine; (2) nornicotine; (3) myosmine; (4) anabasine; (5) anatabine; (6) cotinine. (**B**) The mass spectra of corresponding alkaloids in the chromatogram.

The separation and identification of different alkaloids in *N. tabacum* were performed using GC–MS according to their retention times and confirmed via quadrupole mass spectrometry analysis (Figure 2). Six different alkaloids have been detected in various quantities in leaves among three varieties during five developmental stages. The total alkaloid content ranged from 5.92 to 42.13 mg g^−1^ dry weight (DW), with an average value of 16.57 mg g^−1^ DW (Table 3). The predominant alkaloid was nicotine, representing 93.75% of the total alkaloid content, followed by anatabine (4.12%) and nornicotine (1.67%), whereas the others (myosmine, anabasine, and cotinine) were detected in extremely low amounts.

Hongda, K326 and Zhongyan 100, selected as our plant materials in the current study are three predominant varieties in China and are distributed widely in the corresponding areas, respectively. Alkaloids have been widely investigated in view of being one of the most important secondary metabolites in *N. tabacum* leaves [2,4,7,8,10]. Moreover, its profiles in curing *N. tabacum* leaves have also been reported previously [6,10,11,12]. However, as far as we know, this is the first report addressing the variation in alkaloids among different developmental stages in fresh leaves of three important varieties grown widely in China. In our work, the results demonstrated significant differences in alkaloid composition and content in fresh leaves among different varieties and growth periods. As shown in Table 3, K326 was rich in alkaloids, with the highest level of total alkaloids as well as all individual alkaloids, which was remarkably higher than that of Zhongyan 100 and Hongda the least, which was consistent with the findings of Shi [10] and Lian [12]. However, Shi [10] found that the total alkaloid contents in Hongda at the middle leave maturing stages were 22.80 and 20.05 mg g^−1^ DW, respectively at two producing areas, which was obviously higher than that in our study. Moreover, the alkaloid contents in K326 at the middle leaves maturing stage in the present study (22.20 mg g^−1^ DW) were significantly less than that reported by Lin [11]. (36.31 mg g^−1^ DW), although it was close to those (22.82, 23.52 and 23.45 mg g^−1^ DW) reported by Shi [10]. Such a discrepancy might be due to variations of the cultivation locations and *N. tabacum* leaf situations (before or after modulation) used in different studies.

In addition, the levels of alkaloids in *N. tabacum* leaves increased evidently along with plant growth. The highest contents of alkaloids existed in the upper leaves maturing stage of overall varieties, followed by the middle and lower leaf maturing stages, while low concentrations were observed at the rosette stage and vigorous growing stage (Table 3). The highest contents of total alkaloids and most alkaloids (nicotine, nornicotine, myosmine, anabasine, and anatabine) also occurred in the variety K326 in the upper leaves maturing stage. In contrast, Zhongyan 100 contained the lowest contents of nicotine, nornicotine, myosmine, and total alkaloids in the vigorous growing stage, although there was no significant variance with Hongda at the same growth period.

### 3.2. The Transcription Levels of the Key Structural Genes of Alkaloid Biosynthesis

**Figure 3:**
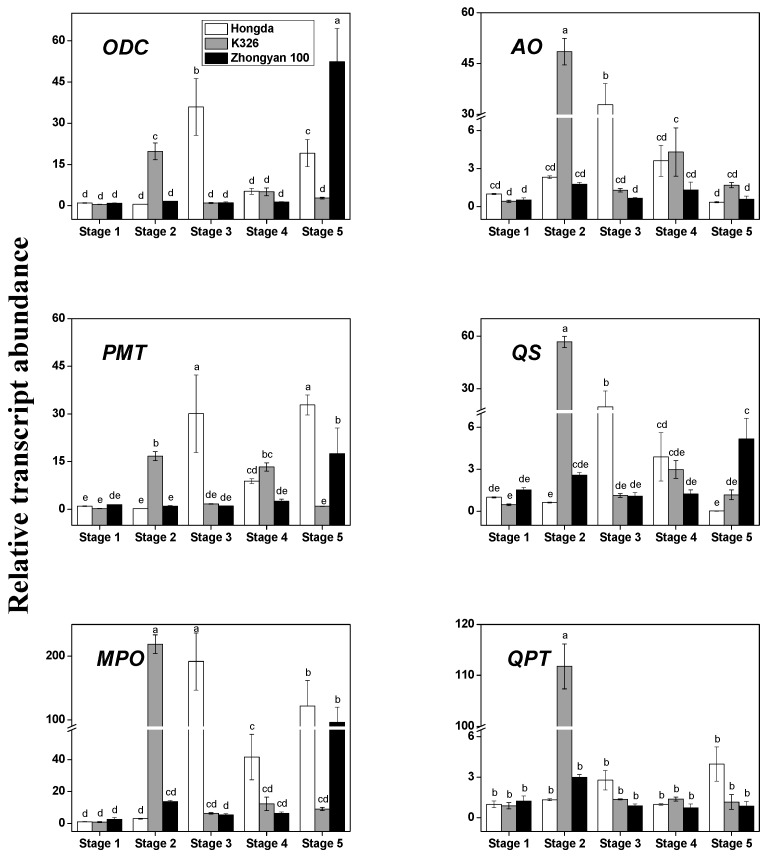
Relative transcript abundance of alkaloid biosynthesis genes in *Nicotiana tabacum* leaves among different varieties and developmental stages. Stage 1, rosette stage; Stage 2, vigorous growing stage; Stage 3, lower leaves maturing stage; Stage 4, middle leaves maturing stage; Stage 5, upper leaves maturing stage. Each value represents the mean (*n* = 3). Values in the same gene followed by the same letter are not significantly different at *p* < 0.05.

Many studies have indicated that alkaloids, especially nicotine, were mainly produced in roots, which was determined by reciprocal grafting experiments, and gathered mostly within the vacuoles of plant roots and leaves [1,7]. However, other studies also reported that the nicotine converted to nornicotine in the leaves, followed by the conversion from nornicotine to another alkaloid in the trichomes [20]. We examined the transcript of several critical genes involved in alkaloid biosynthesis and transportation in *N. tabacum* leaves by qRT-PCR (Figure 3). In the leaves of Hongda, obviously, a high transcription rate for the genes encoding alkaloid biosynthetic enzymes was observed in the lower and/or upper leaf maturing stage. However, gene transcription levels were not generous at the rosette stage and vigorous growing stage. In the leaves of K326, much higher transcription levels of alkaloid-related genes were detected in the vigorous growing stage. Besides, the most abundant transcription levels in Zhongyan 100 leaves were displayed at the upper leaves maturing stage (except AO and QPT). Furthermore, all transcripts of the above-mentioned genes among the three varieties showed remarkable differences. For instance, MPO and QPT levels in K326 leaves at the vigorous growing stage were sharply and significantly increased by 75-fold and 84-fold, respectively, compared with those of Hongda and their transcription levels were 16-fold and 37-fold higher than those of Zhongyan 100, respectively. In the lower leaves maturing stage, an apparent enhancement of the gene transcription levels in Hongda leaves was discovered over that of K326 (by 17.2-fold to 37.3-fold) and Zhongyan 100 (by 18.3-fold to 49.3-fold) in almost all transcripts (except for QPT). Although the transcription levels in Zhongyan 100 in the middle leaves maturing stage were lower than in the other two varieties, the differences among the three varieties were not as obvious as during the two previous periods.

Previous studies reported that the structural genes, such as QPT, in the aerial parts were not related to nicotine biosynthesis [21]. However, an interesting phenomenon found in our study was that the content of alkaloids in tobacco leaves in the leaves maturing stage obviously correlated with the gene transcription levels in leaves in the vigorous growing stage among all three varieties (Figure 3). The correlation analysis was further confirmed by the high correlation between the above two (Table 4), implying that the latter might contribute to the former to some extent. Alkaloid biosynthesis is not a random process but is highly regulated by plant development [1]. Although the content of alkaloids in the vigorous growing stage was low and there was no disparity among varieties but transcription levels of the biosynthetic genes were so different among varieties and might determine the respective content of the alkaloids. It might be the reason for this phenomenon, however, this hypothesis needs further confirmation.

It is reported that nicotine is composed of a pyridine ring and a pyrrolidine ring. Ornithine decarboxylase, PMT, and MPO are involved in the formation of the pyrrolidine ring, whereas some enzymes involved in the early steps of NAD biosynthesis are responsible for the formation of the pyridine ring, such as AO, QS, and QPT (Figure 1) [4,7]. The results in the present study indicated that the transcripts in the upper leaves maturing stage were divided into two categories according to the transcription levels; ODC, PMT, and MPO, were induced prominently and sufficiently, whereas AO, QS, and QPT were entirely decreased after the vigorous growing stage (Figure 3). Such a discrepancy probably contributed to the variations of function and site of action in alkaloid biosynthesis.

### 3.3. Variance Analysis of Genetic Effects

The results of the TestR model in QGAStation (Table 5) showed that considering the genetic effect, the variety, developmental stage, and their interaction effect played major roles in alkaloid accumulation. Plant metabolism was both controlled by genetic and environmental factors [22]. In this study, three varieties were grown at different locations. The most important reason is that each variety needs different environmental conditions and has a respective cultivation area and no single location is suitable for all three varieties simultaneously. Therefore, the variety effect in Table 5 is actually the synthesized effect between each variety and its corresponding environmental factor. Especially, the developmental stage effect was extremely significant at the 0.01 level in all genetic traits. Anatabine and anabasine were mainly controlled by developmental stage effect with the ratio of genetic variance component at 88.7 and 86.5% respectively, which were 18-fold more than that of the variety variance. The developmental stage effect on myosmine was the lowest, whereas its value still occupied almost half of the total genetic variance. The variety effect and interaction effect on each alkaloid were nearly equal to the genetic variance component, except that the interaction variance on nornicotine was 1.5 times higher than the variety one. In contrast, the ratios of error variance were quite low, which indicated that it was barely affected by the genetic effects.

### 3.4. The Correlation among Different Alkaloids

The phenotypic and genotypic correlation coefficients between pairs of nicotine, nornicotine, myosmine, anabasine, anatabine, cotinine, and total alkaloids were extremely significant at the 0.01 level (Table 6), moreover, all traits were positively correlated with each other. The correlation coefficient of nornicotine and anatabine topped others, with the phenotypic correlation of 0.895 and genotypic one of 0.915; the correlation coefficient between anabasine and cotinine were the lowest with a phenotypic correlation of 0.422 and genotypic one of 0.451. The correlation coefficients between cotinine and other alkaloids were lower than those of other pairs and the results implied that cotinine was probably different from other alkaloids in spite of the significant correlation. Similar results were also found by Lian [12] that the phenotypic correlation coefficients between pairs of nicotine, nornicotine, anabasine, anatabine, and total alkaloids were extremely significant at the 0.01 level. However, the correlation coefficients were higher than those in our study, while the highest value was found between nicotine and total alkaloids, instead of between nornicotine and anatabine. Such a difference might be attributed to variations in the developmental stages, varieties and sample forms in different studies. The phenotypic correlation reflects the correlation between the interaction of genotype and other factors and it is usually not in line with the genetic correlation if other effects are taken into consideration [23]. Our results suggested that entire genotypic correlations were higher than their corresponding phenotypic ones (Table 6). Based on the above, there is no doubt that other factors existed and influenced the phenotypic correlation.

To further illuminate the correlation between pairs of alkaloids, the genetic correlation coefficient was divided into different genetic effects (Table 6). Similar to the phenotypic and genotypic correlation coefficients, the variety of correlation coefficients, as well as the developmental stage and interaction was positively and exceedingly significant. The developmental stage correlations of all combinations, with the exceptions of those including cotinine, surpassed the variety and interaction correlations. It was determined that the developmental stage was an indispensable factor for us to research alkaloid metabolism, even exceeding the variety difference. In addition, the top correlation coefficients of variety, developmental stage, and their interaction were obtained for nornicotine with total alkaloids (0.902), anatabine (0.981) and anatabine (0.861), respectively (Table 6).

## 4. Conclusions

In the present study, the composition and content of alkaloids, as well as related gene transcription among the three varieties and five developmental stages, were analyzed. The variety and developmental stage containing the highest alkaloid content were K326 and the upper leaves maturing stage, respectively. Moreover, the alkaloid contents at the leaves maturing stages might be relevant to the transcript of biosynthetic genes in leaves at the vigorous growing stage among the three varieties. To further investigate alkaloid metabolism in *N. tabacum*, the analysis of genetic effects and correlation was also carried out. The results showed that developmental stage played the major role in alkaloid accumulation, and the highest correlation coefficient was shown up in nornicotine with anatabine and total alkaloids, respectively. Taken together, the findings will contribute to the understanding of alkaloid metabolism in fresh leaves during whole plant growth periods and are expected to shed new light on alkaloid biosynthesis and regulation.

## Figures and Tables

**Table 1:** The information about plant material sampling.

Variety	Site	Developmental Stage	Sampling Leaf Position	Sampling Time (yy/mm/dd)
Hongda	Xiangyun county, Yunnan Province, China	Rosette stage	Middle part	11/06/20
		Vigorous growing stage	Middle part	11/07/06
		Lower leaves maturing stage	4–6	11/07/23
		Middle leaves maturing stage	11–13	11/08/09
		Upper leaves maturing stage	16–18	11/08/25
K326	Zunyi county, Guizhou Province, China	Rosette stage	Middle part	11/06/20
		Vigorous growing stage	Middle part	11/07/06
		Lower leaves maturing stage	4–6	11/07/23
		Middle leaves maturing stage	11–13	11/08/09
		Upper leaves maturing stage	16–18	11/08/25
Zhongyan 100	Xiangxian county, Henan Province, China	Rosette stage	Middle part	11/06/20
		Vigorous growing stage	Middle part	11/07/06
		Lower leaves maturing stage	4–6	11/07/23
		Middle leaves maturing stage	11–13	11/08/09
		Upper leaves maturing stage	16–18	11/08/25

**Table 2:** Primer sequences for quantitative real-time polymerase chain reaction analysis.

Gene Name	Forward (F) or Reverse (R)	Sequence	Reference
*ODC*	F	ACTGTGTTTGGGCCCACTTG	[4]
	R	CCATATTAGGAAAAACCAGC	
*PMT*	F	ATTGGACCAAGATCGAGTC	[4]
	R	ATTACTGCAGAATTCTCCTAC	
*MPO*	F	CAGTGATGTTACTGAAACTA	[4]
	R	ATAGGCGAGGAGGACTCATG	
*AO*	F	TTAACAAAGTCATCCGTCGG	[4]
	R	ATTTAGTCTTGAGGTAGACC	
*QS*	F	AATCACTGCTTGATGGTATC	[4]
	R	ACTGGCAAGTTCTTGGACTC	
*QPT*	F	GACGCATTCCGTGAAAGCAC	[4]
	R	AAGTAATGGCGCTCATGCTC	
*26S-RNA*	F	GAAGAAGGTCCCAAGGGTTC	[16]
	R	TCTCCCTTTAACACCAACGG	

**Table 3:** Alkaloid composition and contents in *Nicotiana tabacum* leaves among different varieties and developmental stages.

Variety	Developmental Stage	Nicotine (mg g^−1^ DW)	Nornicotine (μg g^−1^ DW)	Myosmine (μg g^−1^ DW)	Anabasine (μg g^−1^ DW)	Anatabine (μg g^−1^ DW)	Cotinine (μg g^−1^ DW)	Total Alkaloids (mg g^−1^ DW)
Hongda	Rosette stage	7.31 ± 0.35 g	47.12 ± 2.55 f	3.95 ± 0.48 f	6.21 ± 0.73 g	97.38 ± 9.06 h	5.96 ± 1.25 e	7.47 ± 0.36 g
	Vigorous growing stage	6.22 ± 0.49 g	37.77 ± 4.07 f	3.39 ± 0.58 f	8.53 ± 1.25 g	180.58 ± 32.41 gh	4.58 ± 0.40 e	6.46 ± 0.52 g
	Lower leaves maturing stage	7.59 ± 0.66 g	54.03 ± 0.94 f	3.70 ± 0.06 f	10.48 ± 1.00 g	286.95 ± 45.29 g	6.15 ± 1.04 e	7.95 ± 0.66 g
	Middle leaves maturing stage	11.70 ± 0.22 f	124.77 ± 23.88 e	12.22 ± 1.54 de	27.75 ± 2.17 f	607.87 ± 60.02 f	34.39 ± 2.54 c	12.50 ± 0.24 f
	Upper leaves maturing stage	21.85 ± 2.20 cd	321.10 ± 11.40 cd	17.10 ± 3.22 d	76.72 ± 7.22 c	1307.54 ± 126.89 c	8.25 ± 0.49 e	23.58 ± 2.32 cd
K326	Rosette stage	5.77 ± 0.26 g	47.52 ± 2.65 f	4.04 ± 1.69 f	5.63 ± 0.19 g	104.41 ± 8.28 h	5.71 ± 1.95 e	5.94 ± 0.26 g
	Vigorous growing stage	11.34 ± 1.06 f	119.31 ± 20.41 e	9.38 ± 1.64 e	13.35 ± 0.17 g	273.91 ± 39.38 g	7.71 ± 1.21 e	11.76 ± 1.10 f
	Lower leaves maturing stage	22.97 ± 1.69 c	347.05 ± 34.48 c	27.54 ± 2.90 c	33.99 ± 2.69 ef	738.93 ± 38.97 ef	33.05 ± 5.25 c	24.15 ± 1.69 c
	Middle leaves maturing stage	20.75 ± 0.73 d	358.34 ± 16.00 c	36.42 ± 5.64 b	49.28 ± 5.06 d	933.56 ± 187.86 d	72.70 ± 12.28 a	22.20 ± 0.67 cd
	Upper leaves maturing stage	38.82 ± 0.51 a	1055.81 ± 94.16 a	58.41 ± 3.52 a	128.18 ± 8.66 a	2029.32 ± 179.12 a	43.67 ± 2.29 b	42.13 ± 0.72 a
Zhongyan 100	Rosette stage	7.25 ± 0.12 g	49.03 ± 3.81 f	3.82 ± 0.42 f	6.87 ± 0.85 g	102.53 ± 8.67 h	6.28 ± 1.17 e	7.42 ± 0.12 g
	Vigorous growing stage	5.73 ± 0.68 g	46.02 ± 2.48 f	2.46 ± 0.22 f	6.67 ± 0.94 g	133.08 ± 14.44 h	2.45 ± 0.20 e	5.92 ± 0.69 g
	Lower leaves maturing stage	18.11 ± 0.59 e	280.40 ± 52.63 d	16.43 ± 0.97 d	33.27 ± 1.38 ef	702.27 ± 34.30 f	19.01 ± 2.94 d	19.16 ± 0.67 e
	Middle leaves maturing stage	20.29 ± 1.13 d	369.45 ± 15.49 c	23.11 ± 1.52 c	42.67 ± 3.35 de	873.33 ± 60.32 de	42.32 ± 9.34 b	21.65 ± 1.19 d
	Upper leaves maturing stage	27.38 ± 3.12 b	897.02 ± 28.12 b	27.49 ± 8.15 c	104.20 ± 21.69 b	1881.15 ± 67.92 b	25.55 ± 5.22 d	30.32 ± 3.17 b

DW means dry weight. Each value is the mean of three replicates (mean ± standard error). Values in the same column followed by the same letter are not significantly different at *p* < 0.05.

**Table 4:** Correlation coefficient between gene transcription levels at the vigorous growing stage and alkaloids contents in *Nicotiana tabacum* leaves.

Trait	Developmental Stage	*ODC*	*PMT*	*MPO*	*AO*	*QS*	*QPT*
		Vigorous Growing Stage					
Nicotine	Vigorous growing stage	0.991	0.993	0.992	0.998	0.994	0.996
Nornicotine		0.999	0.999	0.999	0.995	0.998	0.997
Myosmine		0.984	0.986	0.986	0.994	0.988	0.991
Anabasine		0.948	0.951	0.95	0.966	0.954	0.959
Anatabine		0.925	0.929	0.928	0.947	0.933	0.939
Cotinine		0.893	0.898	0.897	0.919	0.903	0.91
Total alkaloids		0.991	0.992	0.992	0.997	0.993	0.995
Nicotine	Lower leaves maturing stage	0.777	0.77	0.772	0.736	0.763	0.752
Nornicotine		0.714	0.706	0.708	0.668	0.698	0.686
Myosmine		0.873	0.867	0.868	0.84	0.862	0.853
Anabasine		0.567	0.558	0.56	0.514	0.549	0.534
Anatabine		0.604	0.596	0.598	0.553	0.587	0.573
Cotinine		0.902	0.897	0.899	0.873	0.893	0.885
Total alkaloids		0.772	0.765	0.766	0.73	0.758	0.746
Nicotine	Middle leaves maturing stage	0.582	0.573	0.575	0.53	0.564	0.55
Nornicotine		0.51	0.501	0.503	0.456	0.492	0.476
Myosmine		0.916	0.911	0.912	0.889	0.907	0.899
Anabasine		0.771	0.764	0.766	0.73	0.757	0.754
Anatabine		0.682	0.674	0.676	0.635	0.666	0.653
Cotinine		0.99	0.998	0.988	0.979	0.986	0.983
Total alkaloids		0.586	0.577	0.579	0.534	0.569	0.554
Nicotine	Upper leaves maturing stage	0.963	0.96	0.961	0.944	0.957	0.952
Nornicotine		0.705	0.697	0.699	0.66	0.69	0.677
Myosmine		0.982	0.979	0.98	0.968	0.977	0.973
Anabasine		0.872	0.867	0.868	0.84	0.862	0.853
Anatabine		0.697	0.689	0.691	0.651	0.682	0.669
Cotinine		0.897	0.892	0.893	0.868	0.887	0.879
Total alkaloids		0.951	0.947	0.948	0.93	0.944	0.938

**Table 5:** Estimated proportions of variance components for alkaloids in *Nicotiana tabacum* leaves.

Parameter	Nicotine	Nornicotine	Myosmine	Anabasine	Anatabine	Cotinine	Total Alkaloids
*V_V_*/*V_P_*	0.147 **	0.135 **	0.262 **	0.046 **	0.048 **	0.186 **	0.141 **
*V_D_*/*V_P_*	0.683 **	0.655 **	0.468 **	0.865 **	0.887 **	0.611 **	0.700 **
*V_VD_*/*V_P_*	0.153 **	0.204 **	0.231 **	0.053 **	0.050 **	0.170 **	0.144 **
*V_e_*/*V_P_*	0.018	0.006	0.038 *	0.036	0.016 *	0.033 **	0.016

*V_V_*/*V_P_*: Ratio of variety variance to phenotypic variance. *V_D_*/*V_P_*: Ratio of developmental stage variance to phenotypic variance. *V_VD_*/*V_P_*: Ratio of variety × developmental stage interaction variance to phenotypic variance. *V_e_*/*V_P_*: Ratio of error variance to phenotypic variance. * and ** indicate significance at 0.05 and 0.01 probability levels, respectively.

**Table 6:** Correlation coefficient between pairs of alkaloids in *Nicotiana tabacum* leaves.

Trait	Nicotine	Nornicotine	Myosmine	Anabasine	Anatabine	Cotinine	Total Alkaloids
Nicotine		0.857 **	0.801 **	0.810 **	0.864 **	0.558 **	0.886 **
		0.878 **	0.838 **	0.827 **	0.881 **	0.583 **	0.898 **
Nornicotine	0.900 **		0.775 **	0.843 **	0.895 **	0.483 **	0.868 **
	0.946 **		0.809 **	0.872 **	0.915 **	0.501 **	0.888 **
	0.682 **						
Myosmine	0.832 **	0.809 **		0.698 **	0.750 **	0.657 **	0.807 **
	0.903 **	0.873 **		0.753 **	0.784 **	0.687 **	0.840 **
	0.809 **	0.768 **					
Anabasine	0.818 **	0.851 **	0.768 **		0.877 **	0.422 **	0.821 **
	0.917 **	0.947 **	0.865 **		0.899 **	0.451 **	0.838 **
	0.626 **	0.778 **	0.803 **				
Anatabine	0.803 **	0.865 **	0.741 **	0.774 **		0.487 **	0.876 **
	0.973 **	0.981 **	0.939 **	0.954 **		0.509 **	0.892 **
	0.666 **	0.861 **	0.709 **	0.663 **			
Cotinine	0.851 **	0.817 **	0.854 **	0.780 **	0.734 **		0.557 **
	0.535 **	0.404 **	0.638 **	0.420 **	0.513 **		0.581 **
	0.569 **	0.616 **	0.736 **	0.623 **	0.636 **		
Total Alkaloids	0.828 **	0.902 **	0.831 **	0.820 **	0.805 **	0.848 **	
	0.947 **	0.950 **	0.906 **	0.922 **	0.977 **	0.532 **	
	0.848 **	0.712 **	0.819 **	0.644 **	0.688 **	0.585 **	

The phenotypic correlation coefficient and genotypic correlation coefficient are located in the upper and lower line in the upper right corner of the table, respectively. The variety correlation coefficient, developmental stage correlation coefficient and variety × developmental stage interaction correlation coefficient are located in the upper, middle and lower line in the lower left corner of the table, respectively. * and ** indicate significance at 0.05 and 0.01 probability levels, respectively.
